# Supplementary material for: A discrete region of the D4Z4 is sufficient to initiate epigenetic silencing
Source: Hum Mol Genet. 2025 Jul 8;34(18):1526–40. doi: 10.1093/hmg/ddaf114 (PMC12409625; doi:10.1093/hmg/ddaf114)
Supplement: Paatela_Supplementary_Materials_PDF_ddaf114 [file paatela_supplementary_materials_pdf_ddaf114.pdf]

SUPPLEMENTAL MATERIALS

Supplementary Figures:

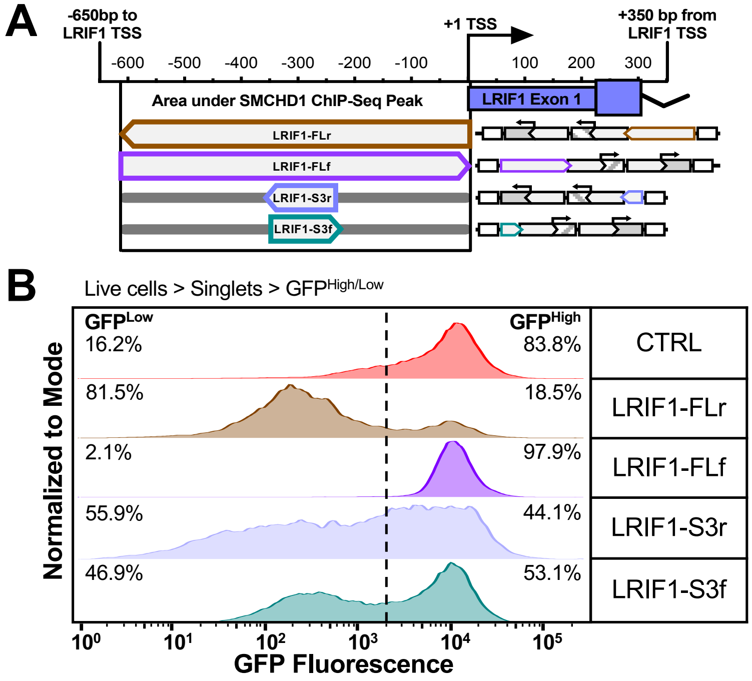

Figure S1: Silencing activity of LRIF1-S3, but not LRIF1-FL, is independent of sequence orientation. (A) Schematic of LRIF1-FLr, LRIF1-FLf, LRIF1-S3r and LRIF1-S3f orientation relative to the genomic sequence and the integrated silencing reporter construct (TSS = transcription start site, bp = base pair). **(B)** Flow cytometry GFP fluorescence histogram of LRIF1-FLr/f and LRIF1-S3r/f compared to CTRL cells, normalized to mode. Note that the higher percentage of silenced cells in the LRIF1-FLr population compared to Fig 1C represents the dynamic increase in silencing over time with the cells shown in this figure at a slightly higher passage number and therefore a higher percentage of silencing.

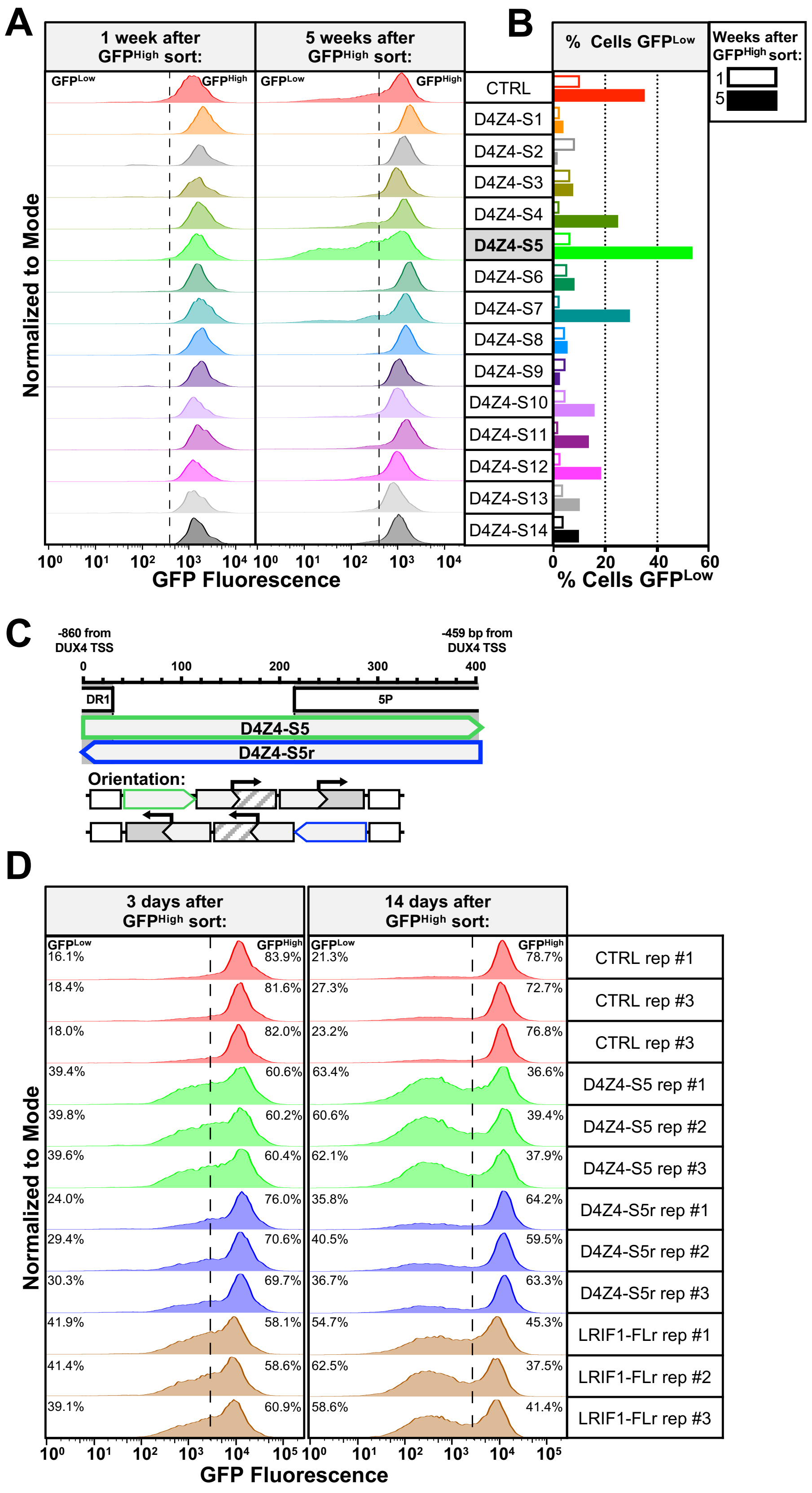

Figure S2: D4Z4-S5 silencing activity is orientation-dependent and increases over time in culture. (A) Flow cytometry GFP fluorescence histogram of D4Z4 silencing construct segments 1 (left) and 5 (right) weeks after GFP^{High} FACS sort, normalized to mode. **(B)** Percentage of GFP^{Low}

cells in each D4Z4 silencing construct line 1 (clear) or 5 (filled) weeks after GFP^{High} FACS sort. **(C)** Schematic of D4Z4-S5 and D4Z4-S5r orientation relative to the genomic sequence and the integrated silencing reporter construct (TSS = transcription start site, bp = base pair). **(D)** Flow cytometry GFP fluorescence histograms of three independent biological replicate (n=3) polyclonal lines at 3 and 14 days after GFP^{High} sort, normalized to mode. Percentages of cell populations classified GFP^{High} or GFP^{Low} are indicated.

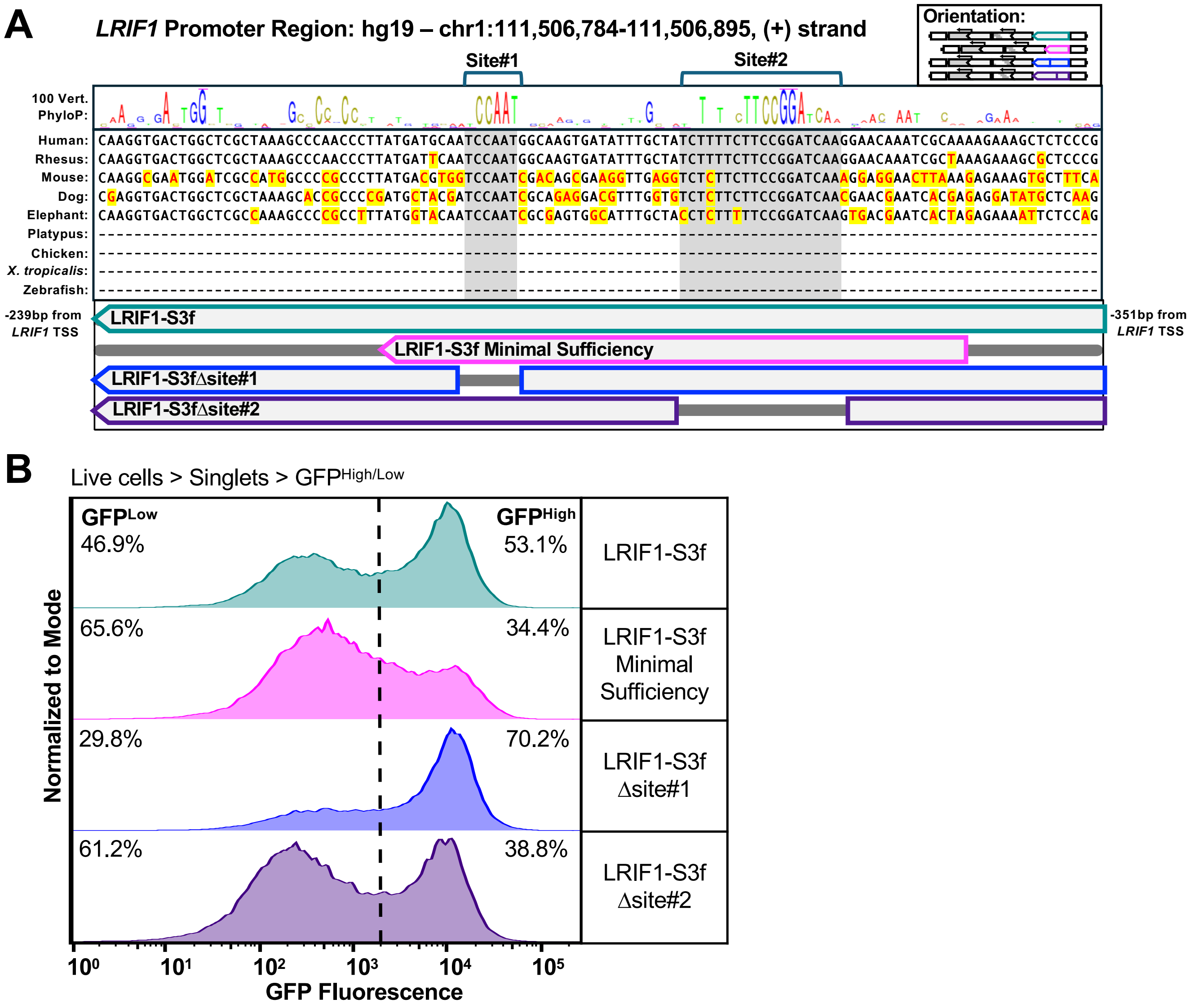

Figure S3: LRIF1-S3 contains a conserved sequence that conveys silencing activity. (A) Top: Logo plot of LRIF1-S3 basewise conservation consensus sequence based on UCSC genome browser phyloP analysis of 100 vertebrates. Middle: LRIF1-S3 sequences in 9 representative species show conservation only in placental mammals. Bottom: Schematic of LRIF1-S3 conserved site deletion inserts with arrowheads indicating orientation relative to genomic sequence and *LRIF1* transcriptional start site (TSS), (bp = base pair). **(B)** Flow cytometry GFP fluorescence histogram of LRIF1-S3 conserved site deletion inserts, normalized to mode. Percentages of cell populations classified GFP^{High} or GFP^{Low} are indicated.

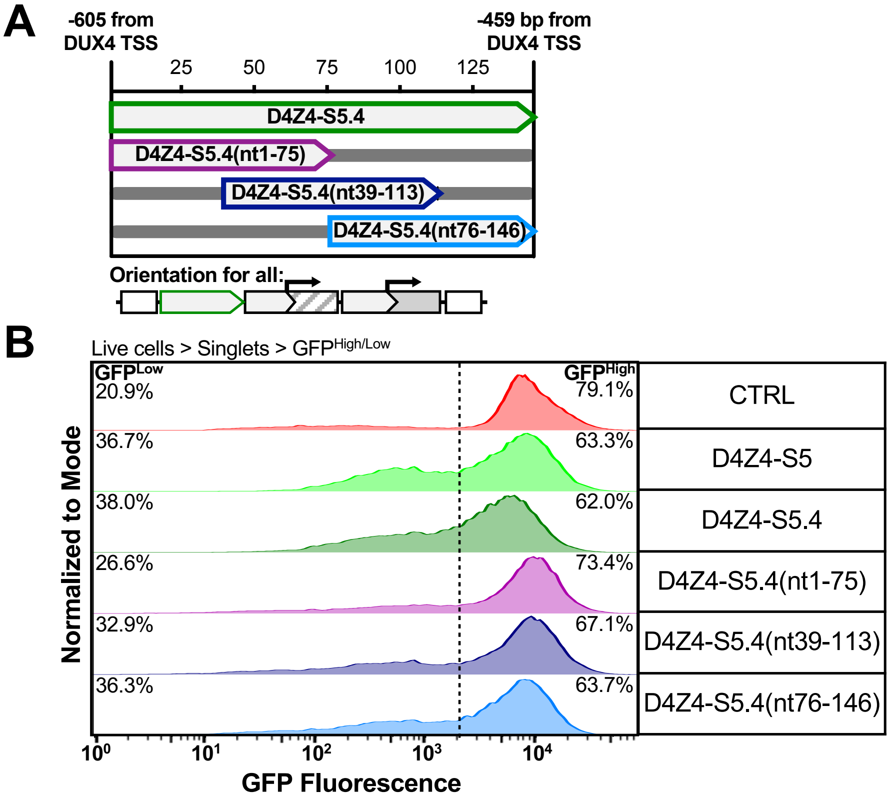

Figure S4: Further segmentation of D4Z4-S5.4 does not narrow region that confers silencing activity. (A) Schematic of D4Z4-S5.4 sub-fragment inserts relative to *DUX4* TSS (nt=nucleotide). **(B)** Flow cytometry GFP fluorescence histogram of D4Z4-S5.4 sub-fragment inserts, normalized to mode. Percentages of cell populations classified GFP^{High} or GFP^{Low} are indicated.

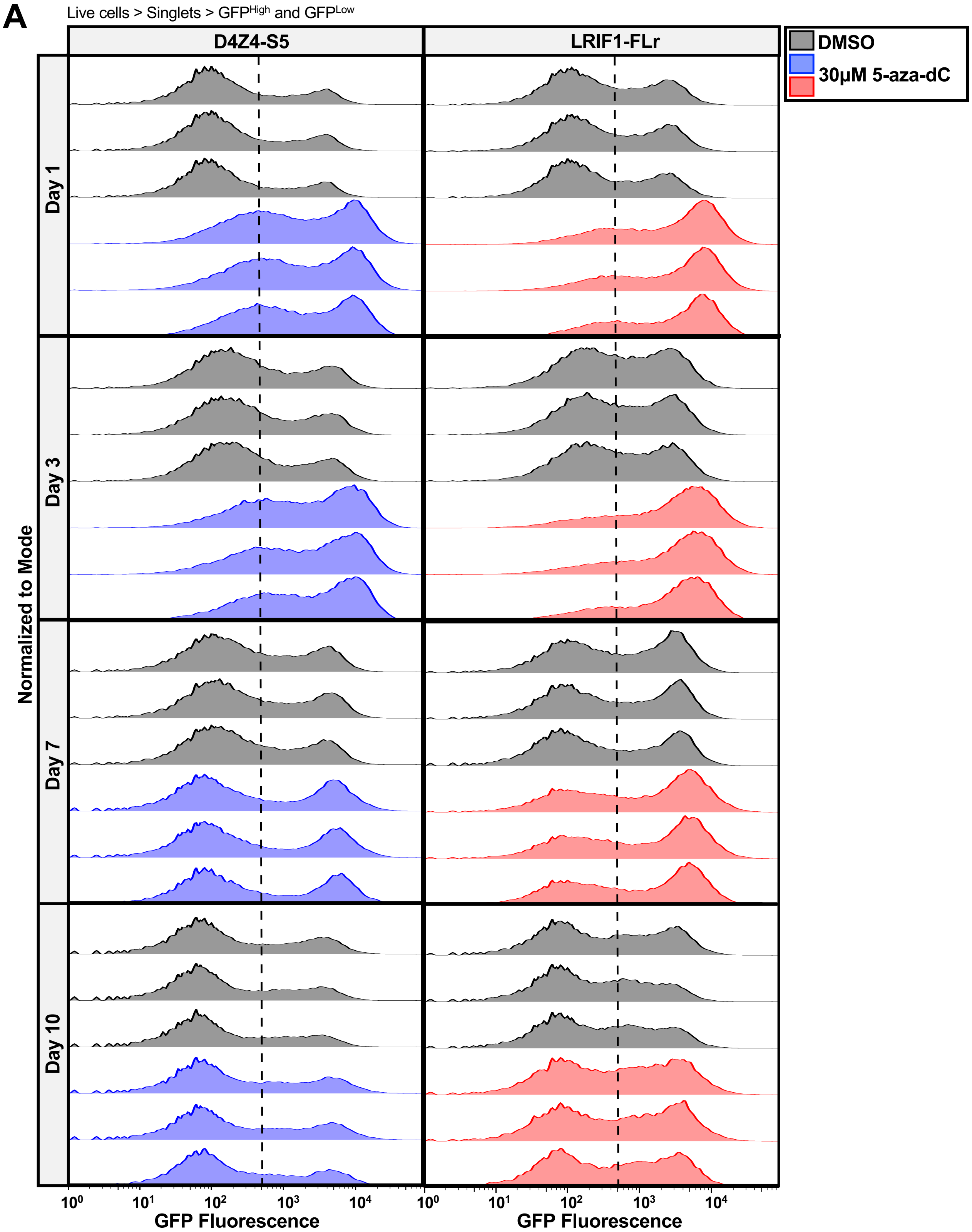

Figure S5: Treatment or D4Z4-S5 or LRIF1-FLr cells with 5aza-dC initially increases GFP expression, followed by GFP re-silencing over time. (A) GFP fluorescence histograms of D4Z4-S5 and LRIF1-FLr cells at various timepoints after 5-aza-dC treatment. Histograms are normalized to the mode of the population. Biological triplicate (n=3) histograms are presented at each time point.

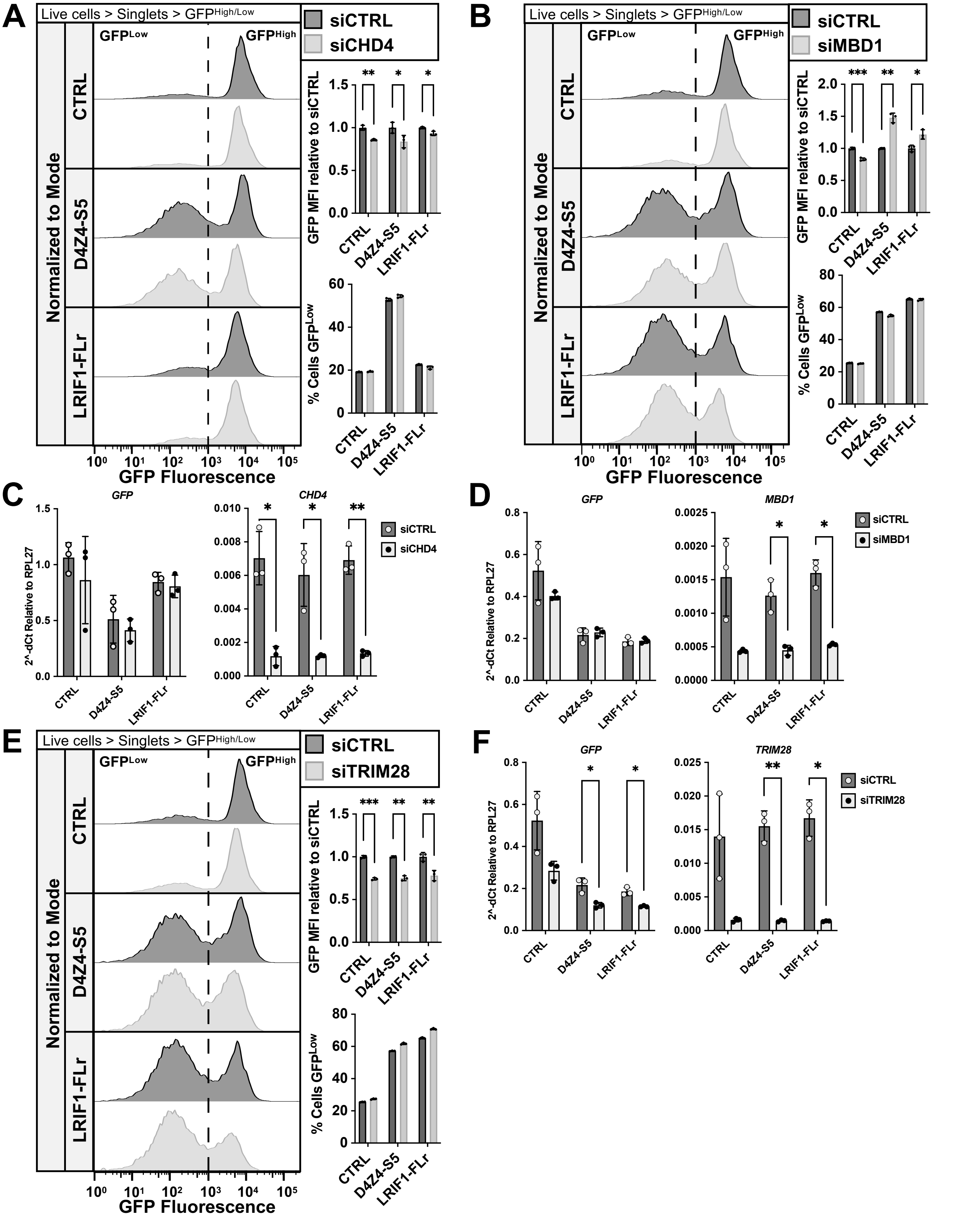

Figure S6: Knockdown of CHD4, MBD1, or TRIM28 does not rescue GFP expression in D4Z4-S5 and LRIF1-FLr cells. (A, B, E) Left: Representative singleton GFP fluorescence histograms of

CTRL, D4Z4-S5 and LRIF1-FLr cells treated with CTRL or CHD4 **(A)**, MBD1 **(B)**, or TRIM28 **(E)** siRNAs. Histograms are normalized to the mode of the population. Right Top: Fold change in GFP median fluorescence intensity (MFI) upon siRNA-mediated knockdown of CHD4 **(A)**, MBD1 **(B)**, or TRIM28 **(E)** compared to siCTRL. Data represent mean ± SD of biological replicates, n=3. Statistical significance was determined by Welch's t-test: *p<0.05, **p<0.01, ***p<0.001. Right bottom: Percentage of GFP^{Low} cells in the population at various time points after siRNA-mediated knockdown of CHD4 **(A)**, MBD1 **(B)**, or TRIM28 **(E)** compared to siCTRL. Data represent mean ± SD of biological replicates, n=3. **(C, D, F)** RT-qPCR analysis of *GFP* (left) and *CHD4* **(C)**, *MBD1* **(D)** or *TRIM28* **(F)** (right) expression compared to housekeeping gene *RPL27* expression in siRNA-treated CTRL, D4Z4-S5, and LRIF1-FLr cells. Data represent mean ± SD of biological replicates, n=3. Statistical significance was determined by Welch's t-test: *p<0.05, **p<0.01.

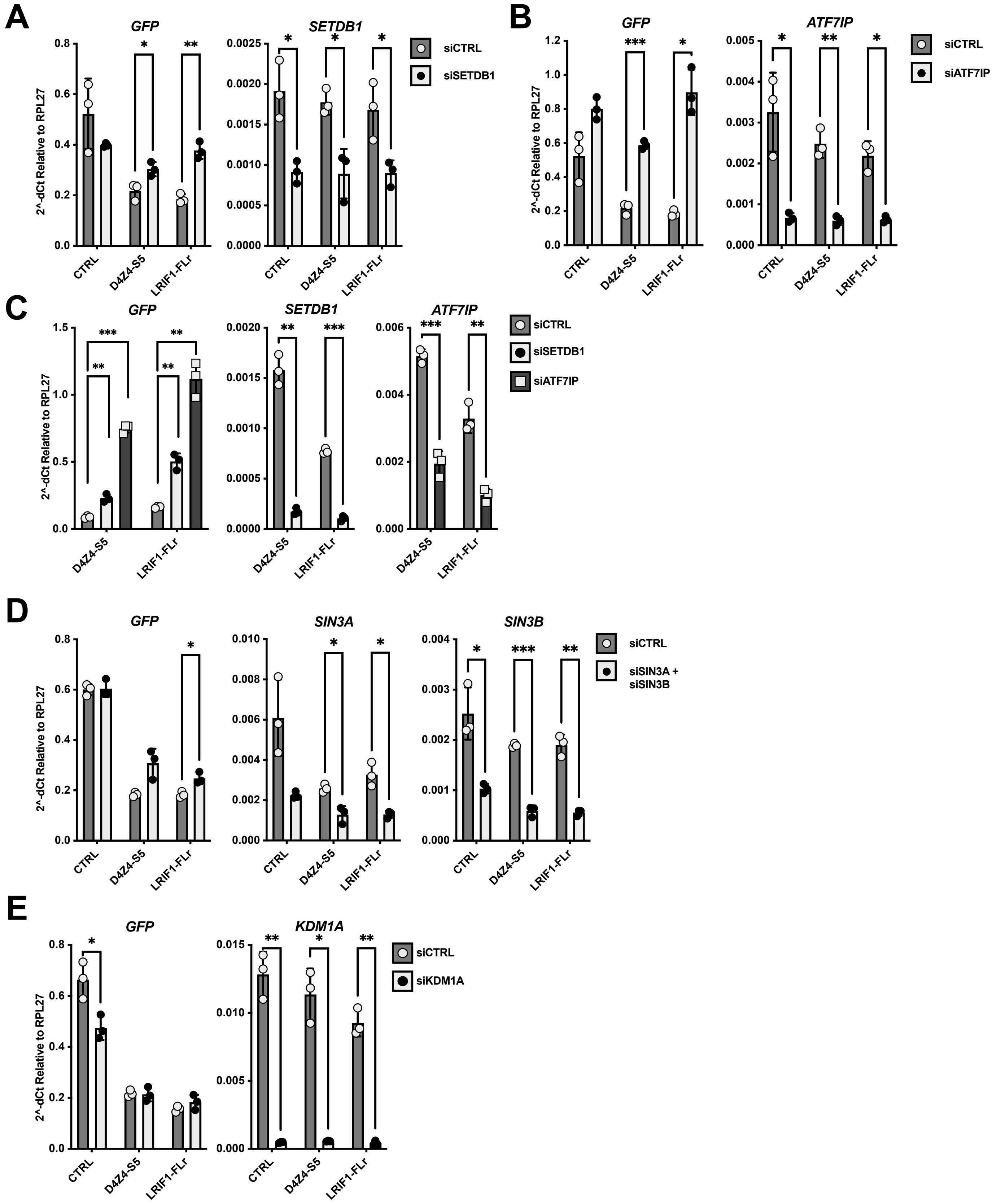

Figure S7: RT-qPCR analysis confirms GFP expression changes and sufficient knockdown efficiency of identified epigenetic regulators. (A-E) RT-qPCR analysis of *GFP* (left) and *SETDB1*

(A), *ATF7IP* **(B)**, *SETDB1 and ATF7IP* from H3K9me3 ChIP samples **(C),** *SIN3A* and *SIN3B* **(D)**, or *KDM1A* **(E)** (right) expression compared to housekeeping gene *RPL27* expression in siRNA-treated CTRL, D4Z4-S5, and LRIF1-FLr cells. Data represent mean ± SD of biological replicates, n=3. Statistical significance was determined by Welch's t-test: *$p<0.05$, **$p<0.01$, ***$p<0.001$.

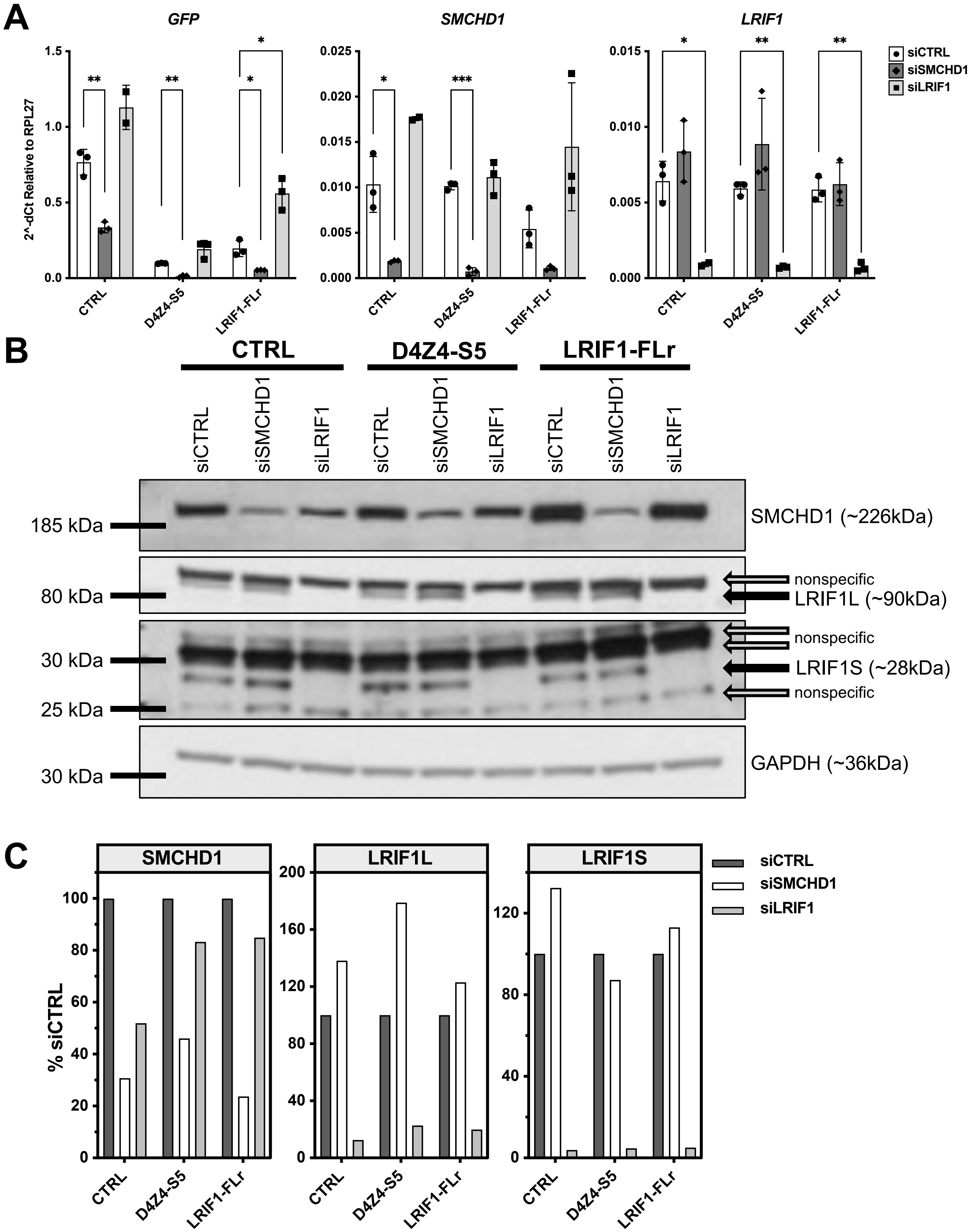

Figure S8: RT-qPCR and western blot analyses confirm changes in GFP expression and sufficient knockdown of SMCHD1 and LRIF1. (A) RT-qPCR analysis of *GFP* (left), *SMCHD1* (middle) and *LRIF1* (right) expression compared to housekeeping gene *RPL27* expression in siRNA-

treated CTRL, D4Z4-S5, and LRIF1-FLr cells. Data represent mean ± SD of biological replicates, n=3. Statistical significance was determined by Welch's t-test: *p<0.05, **p<0.01, ***p<0.001. **(B)** Immunoblot analysis of SMCHD1 and LRIF1 protein levels in CTRL, D4Z4-S5, and LRIF1-FLr cells treated with siCTRL, siSMCHD1, or siLRIF1. Nonspecific band patterns mirror those shown in LRIF1 KO line validation in Šikrová et al., 2023 (38). GAPDH serves as a loading control. **(C)** Quantification of SMCHD1 (left), LRIF1L (middle), and LRIF1 (right) levels in (**B)** using densitometry analysis normalized to GAPDH and graphed as percentage relative to siCTRL-treated samples.

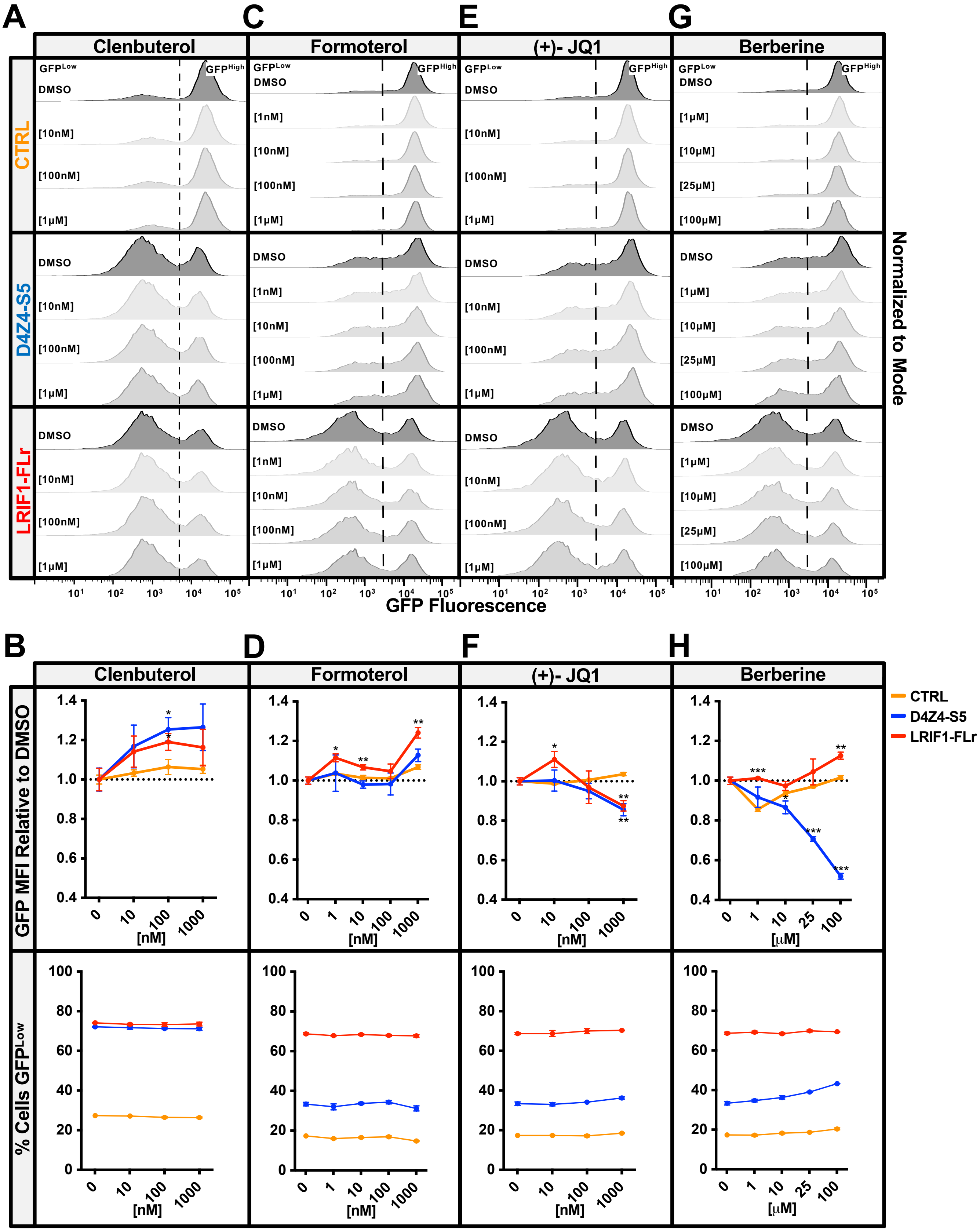

Figure S9: D4Z4-S5 and LRIF1-FLr show differential sensitivity to candidate FSHD therapeutics. (A, C, E, G) Representative singleton GFP fluorescence histograms of CTRL, D4Z4-S5

and LRIF1-FLr cells treated for 3 days with DMSO or escalating dosages of candidate FSHD therapeutics clenbuterol **(A)**, Formoterol **(C)**, (+)- JQ1 **(E)**, or Berberine **(G)**. Histograms are normalized to the mode of the population. **(B, D, F, H)** Top: Fold change in GFP median fluorescence intensity (MFI) upon 3-day treatment with escalating dosages of clenbuterol **(B)**, Formoterol **(D)**, (+)- JQ1 **(F)**, or Berberine **(H)** compared to DMSO treatment in CTRL, D4Z4-S5, and LRIF1-FLr cells. Data points represent mean fold change ± SD of biological replicates, n=3. Statistical significance was determined by Welch's t-test: *$p<0.05$, **$p<0.01$, ***$p<0.001$. Bottom: Percentage of GFP^{Low} cells in the population upon treatment with escalating dosages of clenbuterol **(B)**, Formoterol **(D)**, (+)- JQ1 **(F)**, or Berberine **(H)**. Data represent mean ± SD of biological replicates, n=3.

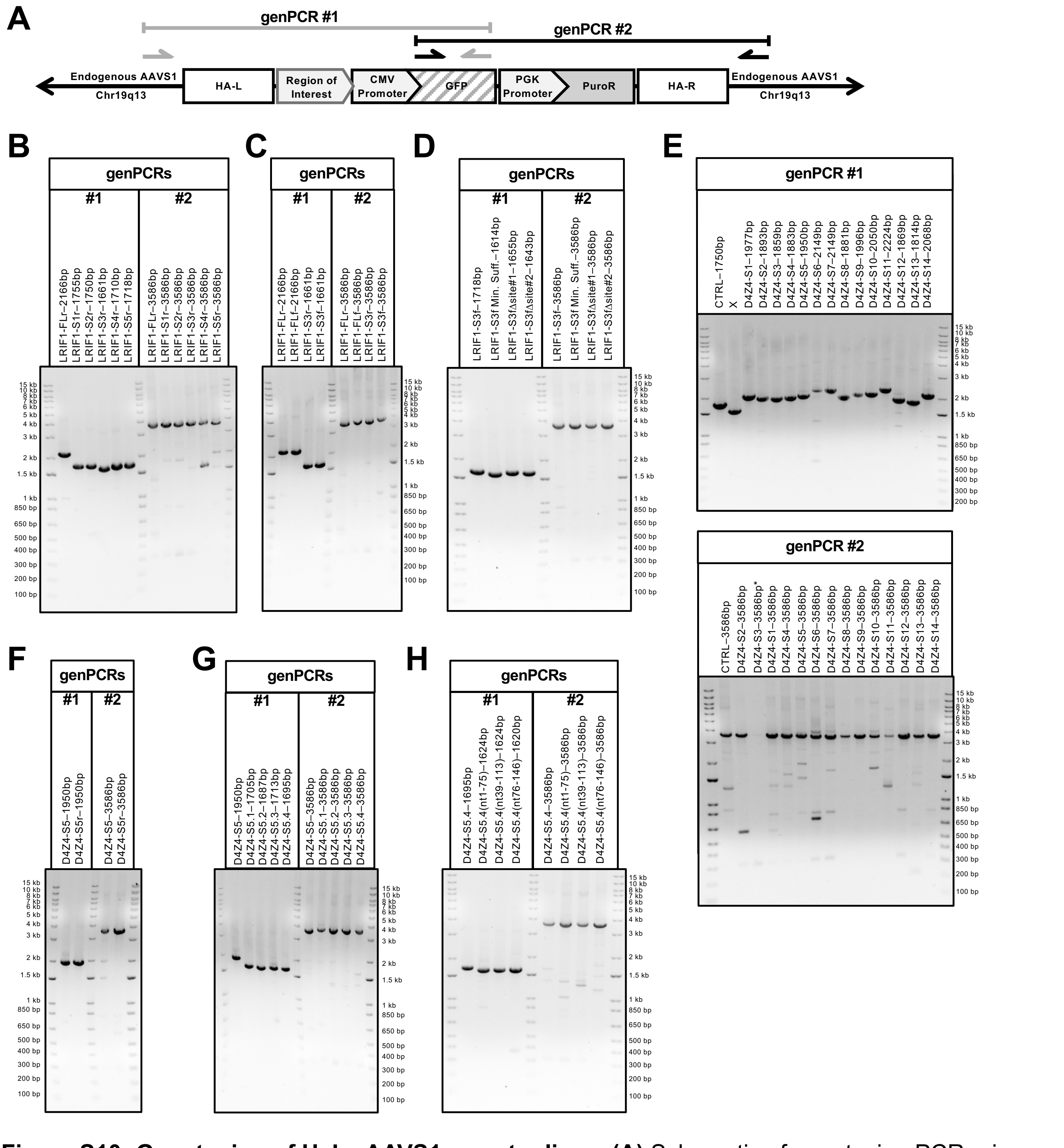

Figure S10: Genotyping of HeLa AAVS1 reporter lines. (A) Schematic of genotyping PCR primer locations. Overlapping PCRs each contain one primer outside the homology arms in the *AAVS1* genomic region to insure correct integration. **(B-H)** Gel electrophoresis indicating size of genotyping PCR products in LRIF1 segment **(B-D)**, D4Z4 segment **(E)**, and D4Z4-S5 sub-segmentation **(F-H)** AAVS1 construct lines. Sequencing validation of CTRL, D4Z4-S5, and LRIF1-FLr lines are shown in Supplemental Material, Table S1. Asterisk (*) indicates low DNA yield limited PCR amplification.

<u>Supplementary Figures:</u>

Table S1: Length, GC, and CpG content of silencing construct inserts and sequencing validation of CTRL, D4Z4-S5, and LRIF1-FLr genotyping PCRs.

Table S2: Individual CpG methylation status based on the average across all sequencing reads.

Table S3: Oligonucleotides, sequences, constructs, reagents and resources used in this study.
